# Impact of COVID-19 on Youth With Type 2 Diabetes: Lessons Learned From a Pediatric Endocrinologist and a Psychologist

**DOI:** 10.3389/fendo.2021.650492

**Published:** 2021-05-27

**Authors:** Cynthia E. Muñoz, Lily C. Chao

**Affiliations:** ^1^ Keck School of Medicine, University of Southern California, Los Angeles, CA, United States; ^2^ Center for Endocrinology, Diabetes, and Metabolism, Children’s Hospital Los Angeles, Los Angeles, CA, United States; ^3^ University Center for Excellence in Developmental Disabilities, Children'’s Hospital Los Angeles, Los Angeles, CA, United States

**Keywords:** COVID-19, pediatric diabetes, type 2 diabetes, SARS-CoV2, psychosocial

## Introduction

The coronavirus disease (COVID-19) continues to devastate the diabetes community. People with type 2 diabetes (T2D) are at significantly higher risk for severe illness if infected by SARS-CoV2 (1, 2). It is unknown if youth with T2D are equally at risk for severe COVID-19. The Type 2 Diabetes Clinic at Children’s Hospital Los Angeles serves more than 450 youth with type 2 diabetes. From the perspectives of a pediatric endocrinologist and psychologist, we have witnessed the physical and social-emotional impact of this pandemic on youth with T2D and their families.

## Physical Risks for Youth With T2D

The literature abounds with studies demonstrating diabetes, obesity, and hypertension as independent risk factors for severe COVID-19 infection (1, 3, 4). Unless explicitly stated, references to “diabetes” refer presumably to T2D, given its relative prevalence compared to other types of diabetes. To date, investigations have largely focused on the impact of SARS-CoV-2 on adults, with a paucity of information in youth. We recently showed that the incidence of youth who presented with diabetic ketoacidosis (DKA) when newly diagnosed with T2D nearly doubled since the start of the pandemic (5). Future multi-site studies will be needed to examine if our observation is generalizable to other centers that serve a large population of pediatric T2D. What remains unreported, is the impact of the pandemic on the physical health of youth with existing T2D.

Several characteristics and co-morbidities of youth with T2D increase their potential risk for severe COVID-19 infection. Pediatric T2D in the United States disproportionately affects ethnic and racial minority groups including Latinx and Black communities (6). A similar trend has been detected among people infected with COVID-19 (6, 7). Many youth with T2D develop uncontrolled hyperglycemia 12 to 18 months after they are diagnosed with diabetes (8). In some reports, hyperglycemia in individuals with T2D is associated with increased COVID-19 disease severity and mortality (9, 10). Most youth with T2D have additional co-morbidities, most commonly obesity and hypertension. If infected with SARS-CoV-2, these risk factors may increase their potential to develop severe COVID-19 illness.

Beyond the threat of SARS-CoV2 infection, the pandemic has adversely impacted the physical health of youth with T2D in our clinical practice. School closures have eliminated the benefits of school-based health care providers, who served as a critical safety net for many of our patients. For some, the lack of adult supervision has led to worsened medication adherence, poorly managed or unmanaged diabetes, and the development of diabetic ketoacidosis (DKA). The Stay-at-Home order, loss of access to physical education in school, and increased screen time further limits physical activity for these youth. Even though physical education has resumed in a virtual format for some, students who reside in apartments or multi-family homes frequently do not participate due to fear and/or embarrassment of disturbing their neighbors or housemates. In addition, many youths whose access to food was dependent on meals and snacks provided at school have increased their consumption of low-quality nutrition during this time. The lack of physical activity and increased caloric consumption has led to excessive weight gain and worsened glycemic control (11). Together, these factors may exacerbate existing risks for COVID-19 illness for these youth.

The global pandemic has also affected medical access for many pediatric patients with T2D. Although our hospital transitioned to telemedicine soon after the Stay-at-Home order was enacted, many of our patients’ families have had difficulty accessing telehealth due to limited internet connectivity and/or technology literacy. In addition, most of our patients have state-subsidized insurance plans that require intermittent document updates based on in-person assessments. The closure of many government offices for in-person services and an overwhelmed telephone system has limited some families’ ability to keep their insurance requirements up-to-date. In turn, the loss of health insurance benefits has contributed to delays in routine follow-up diabetes care visits and gaps in access to prescribed medications. The fear of exposure to COVID-19 associated with the use of public transportation or with the perceived risk in coming to the hospital has also contributed to missed or rescheduled appointments. As well, diabetes care visits have been postponed due to either the patient or family members testing positive for SARS-CoV2. The delay in accessing routine diabetes care has prevented timely medical intervention in glycemic management placing these youth at greater risk for poorly managed blood glucose and DKA.

## Psychosocial Risks for Youth With T2D

Youth with T2D are at higher risk for depression and anxiety compared to those without diabetes (12, 13). They often experience shame and embarrassment about their medical diagnosis due to stigma. Coupled with frequent reports of teasing and bullying, especially for those with overweight, youth with T2D are at risk for mood disorders and related symptoms such as social isolation and decreased energy and motivation. The COVID-19 pandemic has created additional stressors that compound existing psychosocial challenges for individuals with T2D. Together, these adversities can negatively impact diabetes management behaviors (14, 15).

With the economic downturn, many parents and guardians have reported job loss and/or a reduction in wages. The financial toll has resulted in a cascade of problems including homelessness or fear of eviction and food insecurity. For some, the financial strain has resulted in the fear of job loss by requesting time off and difficulty accessing transportation. These challenges have limited patient access to prescribed treatments including medical appointments and mental health services. Diminished access to this much-needed health care has exacerbated worries and feelings of helplessness and have contributed to the lack of appropriate care for an already vulnerable patient population. Youth experiencing heightened mood symptoms report struggles to follow prescribed diabetes management behaviors leading to a decrease in physical activity, increase in food consumption including unhealthy foods, and decreased motivation to take prescribed medications. Moreover, feelings of uncertainty associated with continuous public health warnings about the COVID-19 pandemic have fueled existing emotional vulnerabilities.

Our pediatric T2D community reports mounting stress amidst the ongoing uncertainties of the pandemic. Many are grieving the death of loved ones who succumbed to complications associated with COVID-19 illness. The youth with T2D and their families we serve report fears about exposure or re-exposure to SARS-CoV-2. Those who previously recovered from COVID-19 wonder if they will have the fortune to recover from mild or moderate symptoms if exposed to the virus again. Fears about risk for exposure lead to social isolation. In turn, some youth with T2D have reported that chronic social isolation during the pandemic has exacerbated feelings of sadness and loneliness. Youth also report worrying about their parent or guardian’s risk for exposure to the virus, given that many adults in this community are essential workers and do not have the luxury to work from home. Despite the fear that they will be exposed to SARS-CoV-2 at work and subsequently exposure their child to this virus, many parents and guardians believe that they have no choice but to work to avoid job loss.

The closure of in-person schooling has also adversely impacted the mental health of our youth. Many youths benefit from school-based services including mental health support. While telehealth options are available, access is significantly limited for families who struggle to access virtual platforms. Some youth are grateful for a break from the potential for negative social interactions within the school environment; however, they also report less opportunities for peer and academic support as well as everyday distractions. Furthermore, youth who prefer in-person and tactile educational opportunities report feelings of frustration, helplessness, and concern about their decline in academic performance. While some schools are resuming in-person educational opportunities, some families feel hesitant about returning to school due to concern around safety protocols and fear of SARS-CoV-2 exposure.

## Conclusions and Recommendations

The COVID-19 pandemic has far-reaching impacts in the care of youth with T2D. The demographic and co-existing co-morbidities may increase their risk for severe COVID-19 infection. While there continues to be much uncertainty about the course and sequelae of this virus, we must continue to assess and respond to the compounding physical and social-emotional impact of this pandemic on pediatric T2D populations. Furthermore, the disruption of the lockdown on the physical health and mental health of these youth must be considered in treatment planning as well as the development of public health policies.

To better understand the needs of youth with T2D, we reiterate the recommendations proposed by DiMeglio et al. (**Table 1**) (16). Specifically, improved data collection for diverse pediatric populations with T2D is needed to better understand the trends and outcomes for this population. It is not sufficient or acceptable to extrapolate from data gathered from adults. Careful interpretation of pediatric T2D data is critical to ensure that appropriate guidelines and precautions are in place within childcare and school settings, and for other activities involving physical interaction.

**Table 1:** Pediatric type 2 diabetes during COVID-19*.

NEEDS	RECOMMENDATIONS
Prospectively-collected, longitudinal, retrievable public data on new onset incidence trends, new onset patient characteristics, and acute and long-term disease outcomes	Collect data for youth with type 2 diabetes following established guidelines for epidemiologic data collections
	Examine effects of obesity and hypertension on COVID-19 severity among youth with T2D
	Improve data collection for racial and ethnic minority populations
Thoughtful interpretations of emerging data reports, including thorough peer review	Contextualize data to inform childcare decisions, including return to school and extracurricular
	Provide a clear and unified voice for pediatric-specific type 2 diabetes concerns at the level of national and international organizations and public health messaging
Access to multidisciplinary care and support including mental health, social work, and case management access	Screen and provide appropriate support resources that target depression, food insecurity, financial stability Support the use of accessible virtual platforms and alternate ways to provide youth with T2D with medical and mental health care in addition to any service required to sustain insurance benefits

*Adapted sections with permission (18).

Psychosocial distress coupled with the chronic uncertainties of the COVID-19 pandemic can have a negative and significant impact on diabetes management behaviors and access to prescribed treatments for youth with T2D. Screening patients’ mental health and basic necessities by health care providers and educators, in addition to multidisciplinary treatment planning, is necessary to address these needs.

## Author Contributions

All authors listed have made a substantial, direct, and intellectual contribution to the work and approved it for publication.

## Conflict of Interest

The authors declare that the research was conducted in the absence of any commercial or financial relationships that could be construed as a potential conflict of interest.
